# Supplementary material for: Splice-Junction-Based Mapping of Alternative Isoforms in the Human Proteome
Source: Cell Rep. Author manuscript; Available in PMC 2020 Jan 15. (PMC6961840; doi:10.1016/j.celrep.2019.11.026)
Supplement: 2 [file NIHMS1546469-supplement-2.zip › DF1/PXD010154/Heart/Heart_2_TNS1_TPLSALGLKPHNPADILLHPTGEEDEGKVVVR.pdf]

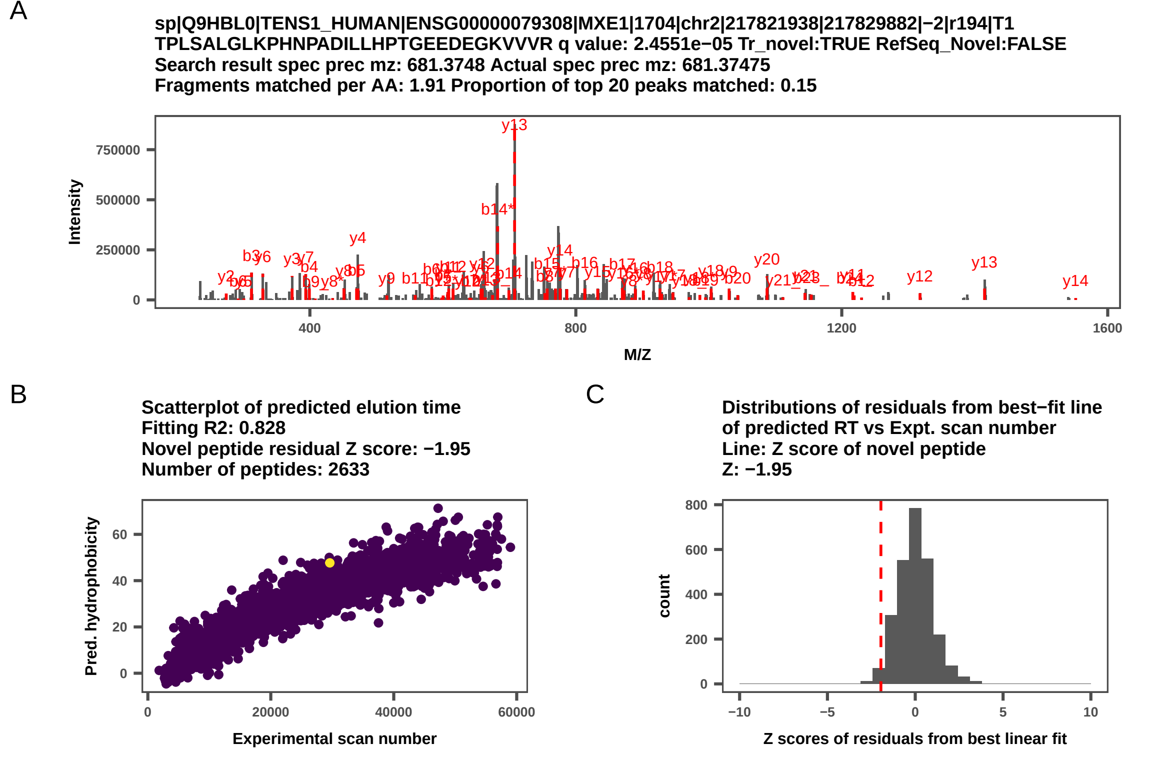
A
sp|Q9HBL0|TENS1_HUMAN|ENSG00000079308|MXE1|1704|chr2|217821938|217829882|−2|r194|T1
TPLSALGLKPHNPADILLHPTGEEDEGKVVVR q value: 2.4551e−05 Tr_novel:TRUE RefSeq_Novel:FALSE
Search result spec prec mz: 681.3748 Actual spec prec mz: 681.37475
Fragments matched per AA: 1.91 Proportion of top 20 peaks matched: 0.15
Intensity
750000
500000
250000
0
y13
b14*
y4
y14
y20
y13
y12
y14
400
800
1200
1600
M/Z
B
Scatterplot of predicted elution time
Fitting R2: 0.828
Novel peptide residual Z score: −1.95
Number of peptides: 2633
Pred. hydrophobicity
60
40
20
0
0
20000
40000
60000
Experimental scan number
C
Distributions of residuals from best−fit line
of predicted RT vs Expt. scan number
Line: Z score of novel peptide
Z: −1.95
count
800
600
400
200
0
−10
−5
0
5
10
Z scores of residuals from best linear fit
